# Clade-specific variation in susceptibility of *Candida auris* to broad-spectrum ultraviolet C light (UV-C)

**DOI:** 10.1017/ice.2020.410

**Published:** 2020-12

**Authors:** Piyali Chatterjee, Hosoon Choi, Brennan Ochoa, Gennifer Garmon, John D. Coppin, Yonhui Allton, Janell Lukey, Marjory D. Williams, Dhammika Navarathna, Chetan Jinadatha

**Affiliations:** 1 Department of Research, Central Texas Veterans’ Health Care System, Temple, Texas; 2 Department of Medicine, Central Texas Veterans’ Health Care System, Temple, Texas; 3 Infectious Diseases, Baylor Scott and White Memorial Healthcare, Temple, Texas

## Abstract

**Background:**

*Candida auris is* an emerging and often multidrug-resistant fungal pathogen with an exceptional ability to persist on hospital surfaces. These surfaces can act as a potential source of transmission. Therefore, effective disinfection strategies are urgently needed. We investigated the efficacy of ultraviolet C light (UV-C) disinfection for *C. auris* isolates belonging to 4 different clades.

**Methods:**

In vitro testing of *C. auris* isolates was conducted using 10^6^ colony-forming units (CFU) spread on 20-mm diameter steel carriers and exposed to a broad-spectrum UV-C light source for 10, 20, and 30 minutes at a 1.5 m (5 feet) distance. Post-UV survivors on the coupons were subsequently plated. Colony counts and log reductions were recorded, calculated, and compared to untreated control carriers. Identification of all isolates were confirmed by MALDI-TOF and morphology was visualized by microscopy.

**Results:**

We observed an increased susceptibility of *C. auris* to UV-C in 8 isolates belonging to clades I, II and IV with increasing UV exposure time. The range of log kill (0.8–1.19) was highest for these isolates at 30 minutes. But relatively no change in log kill (0.04–0.35) with increasing time in isolates belonging to clade III were noted. Interestingly, *C. auris* isolates susceptible to UV-C were mostly nonaggregating, but the isolates that were more resistant to UV exposure formed aggregates.

**Conclusions:**

Our study suggests variability in susceptibility to UV-C of *C. auris* isolates belonging to different clades. More studies are needed to assess whether a cumulative impact of prolonged UV-C exposure provides additional benefit.

*Candida auris*, first reported in 2009, is an emerging fungal pathogen that is often multidrug resistant, difficult to identify, and has an ability to persist in the hospital environment.^1-3^ Also, *C. auris* has been reported to cause hospital outbreaks with high mortality rates, especially among critically ill patients.^4^ The emergence and detection of *C. auris* on multiple continents simultaneously has led to identification of 4 distinct clonal lineages via whole-genome sequencing: clade I (South Asian), clade II (East Asian), clade III (African), and clade IV (South American).^5^ Interestingly, despite intraclonal heterogeneity, very few single-nucleotide polymorphism (SNP) differences exist within each clade. In addition, it has been illustrated that some isolates form aggregates potentially conferring protection from the environment.^6^

Presently, no specific guidelines has been established for *C. auris* disinfection on hospital surfaces. The Centers for Disease Control and Prevention (CDC) recommends the use of Environmental Protection Agency (EPA)–registered hospital-grade disinfectants that are effective against *Clostridioides difficile* (*C. diff*) spores. Surface disinfection protocols with chemical disinfectants have shown variable outcomes.^7,8^ Therefore, to prevent the transmission of *C. auris* from hospital surfaces, more effective strategies in conjunction with daily chemical disinfection of patient-occupied rooms are urgently needed.

Mobile, automated broad-spectrum ultraviolet-C light (UV-C) decontamination systems using pulsed-xenon (PX-UV) or other mercury-based sources have reduced the recovery of methicillin-resistant *Staphylococcus aureus* (MRSA), *Clostridioides difficile*, and vancomycin-resistant *Enterococci* (VRE) from glass carriers and frequently touched surfaces in hospitals.^9^ *Candida* spp, including *C. auris,* appear to be significantly less affected by UV-C decontamination than MRSA.^10^ A recent study demonstrated that UV-C has been effective at reducing colony counts of *C. auris,* especially with longer exposure time and closer proximity to UV source.^11^ However, whether there are notable differences between isolates belonging to different clades in their response to UV-C exposure at increasing time intervals remains unknown. We further evaluated whether the susceptibility to UV-C depends on the aggregate-forming capability of *C. auris* clade(s).

## Methods

We evaluated the efficacy of a pulsed-xenon (PX) UV-C room decontamination device (Xenex Disinfection, San Antonio, TX) against 10 *C. auris* isolates. These 10 isolates of *C. auris* (AR# 0381-0390) were obtained from the CDC & FDA Antibiotic Isolate Bank. The PX-UV device contained a xenon gas flash bulb that operates at 2 Hz and emits a broad spectrum of radiation covering the UV-C spectrum of 200–280 nm as well as the visible light spectrum. For each pathogen, 10 µL aliquots (3 biological replicates) containing 10^6^ colony-forming units (CFU) in phosphate-buffered saline (PBS) with 5% fetal bovine serum (FBS) were spread over 20 mm diameter steel coupons and allowed to air dry for 30 minutes in a laminar flow hood. The steel coupons were then placed perpendicular to the PX-UV lamps of the device and at distance of 1.5 m (5 feet). A set of three biological replicates were exposed to UV-C at 10, 20, and 30 minutes, respectively. Our control groups were not exposed to UV-C and were plated last to account for any desiccation. To quantify viable organisms, treated and untreated control coupons were submersed in 10 mL PBS and vortexed vigorously, and serial dilutions were plated on Sabouraud dextrose agar (SDA, Remel, Lenexa, KS) and were incubated at 37°C for 72 hours. Colonies were then counted, and log reductions were calculated. The experiments were repeated 3 times (technical replicates) to mitigate any variability of the protocol or day-to-day weather conditions such as humidity or airflow.

We used the method of Borman et al^6^ for aggregation assay. First, each *C. auris* isolate was grown on a Sabouraud dextrose agar plate at 37°C for 48 hours. Two closely related yeast strains, such as *C. krusei* (AR#0397) and *C. lusitiniae* (AR# 0398), were used as controls. A cell suspension was prepared with a *C. auris* colony from each of the 10 isolates and vortexed for 30 seconds. Each isolate was resuspended in PBS to achieve a McFarland standard of 4–5 (for a final cell density of 1×10^8^ cells/mL). Then, 30 μL of this suspension was loaded on a glass slide and examined under a microscope (Olympus BX53 laden with Olympus camera DP73).

### Statistical methods

Raw plate data of counts of colony-forming units killed were converted to a log value, and log kill was determined from the initial concentration at time zero. Mean log kill and standard deviations were calculated for each isolate at 10, 20, and 30 minutes. A Bayesian linear regression model with a hierarchical structure for clade, isolate number, and experiment was used to estimate the slope of log kill over time for each clade. Results are reported as the estimated slope of log kill per minute with 95% uncertainty intervals. All analyses were conducted in R version 3.5.3 software (R Foundation for Statistical Computing, Vienna, Austria) utilizing the ‘brms’ package for modeling and the ‘ggplot2’ package for plotting.

## Results

**Fig. 1.**
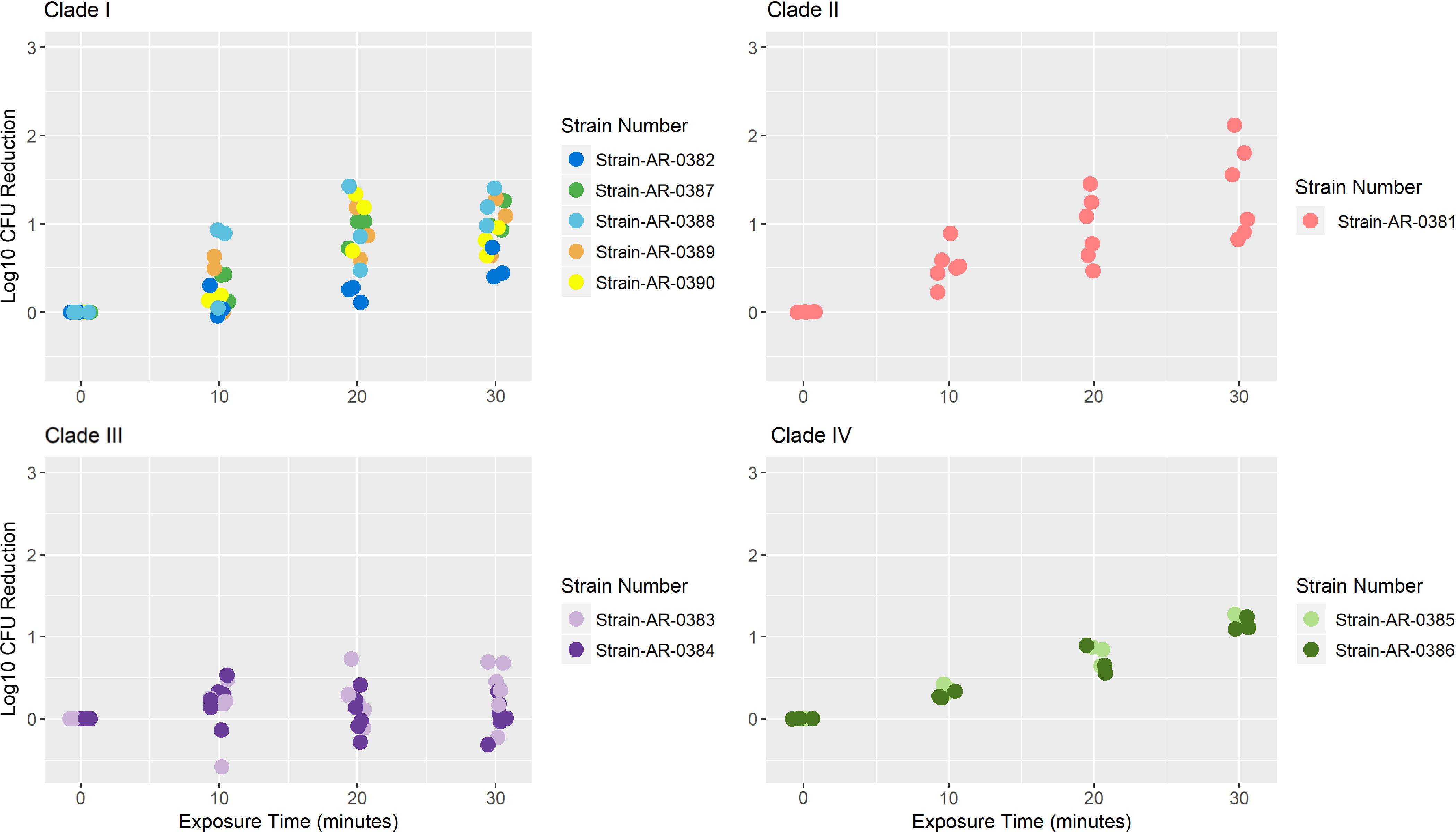
The effect of UV-C exposure on different clades of *C. auris*. Log reductions for each of the *C. auris* isolates belonging to clades I-IV are shown here. Log reductions were calculated by subtracting viable organisms recovered after exposure to UV versus controls (no UV exposure) for each time points (10, 20, 30 minutes).

Mean log kill at 10, 20, and 30 minutes for each of the 10 *C. auris* isolates are shown in Table 1. The raw mean log-kill at 30 minutes for clade 1 was 0.92 (SD, 0.31), for clade II was 1.38 (SD, 0.53), for clade III was 0.19 (SD, 0.32), and for clade IV was 1.18 (SD, 0.07). Also, 5 clade I isolates (AR#0382, AR#0387, AR#0388, AR#0389, AR#0390) were tested with 0.5 log_10_ (AR#0382) to 1.2 log_10_ (AR#0388) reduction at 30 minutes of exposure. One clade II isolate AR#0381 was tested, and it demonstrated a 1.38 log_10_ reduction at 30 minutes of UV-C exposure. Two clade III isolates (AR#0383 & AR#0384) were tested with 0.04 (AR#0384) to 0.35 (AR#0383) log_10_ reduction at 30 minutes of UV-C exposure, showing the worst response to UV-C exposure among all *C. auris* clades. Further, the 2 clade IV strains tested, AR#0385 and AR#0386, showed almost similar reductions in CFU count after UV-C exposure (1.22 and 1.15, respectively). The log-kill data were plotted for each clade and were colored by isolates (Fig. 1).

**Table 1.** Mean Log-Kill at 10, 20, and 30 Minutes for Each *Candida auris* Strain

*C. auris* Clade	Strain No.	10 Minutes, Mean Log Kill (SD)	20 Minutes, Mean Log Kill (SD)	30 Minutes, Mean Log Kill (SD)
Clade I	Strain-AR-0382	0.10 (0.18)	0.21 (0.09)	0.52 (0.18)
Clade I	Strain-AR-0387	0.32 (0.17)	0.93 (0.17)	1.06 (0.18)
Clade I	Strain-AR-0388	0.62 (0.50)	0.92 (0.48)	1.19 (0.21)
Clade I	Strain-AR-0389	0.37 (0.34)	0.88 (0.30)	1.01 (0.33)
Clade I	Strain-AR-0390	0.12 (0.09)	1.07 (0.34)	0.80 (0.16)
Clade II	Strain-AR-0381	0.53 (0.22)	0.94 (0.38)	1.38 (0.53)
Clade III	Strain-AR-0383	0.12 (0.36)	0.25 (0.28)	0.35 (0.35)
Clade III	Strain-AR-0384	0.23 (0.22)	0.06 (0.25)	0.04 (0.22)
Clade IV	Strain-AR-0385	0.36 (0.05)	0.79 (0.12)	1.22 (0.06)
Clade IV	Strain-AR-0386	0.29 (0.04)	0.70 (0.17)	1.15 (0.08)

The model estimated slope of the log kill over time pooled across all clades and isolates was 0.03 (−0.004 to 0.06) log-kill per minute of UV exposure. The model-estimated slopes of the log kill per minute of UV over time were 0.03 (0.02–0.05) for clade I, 0.04 (0.02–0.06) for clade II, 0.01 (−0.004 to 0.03) for clade III, and 0.04 (0.02–0.06) for clade IV.

**Fig. 2.**
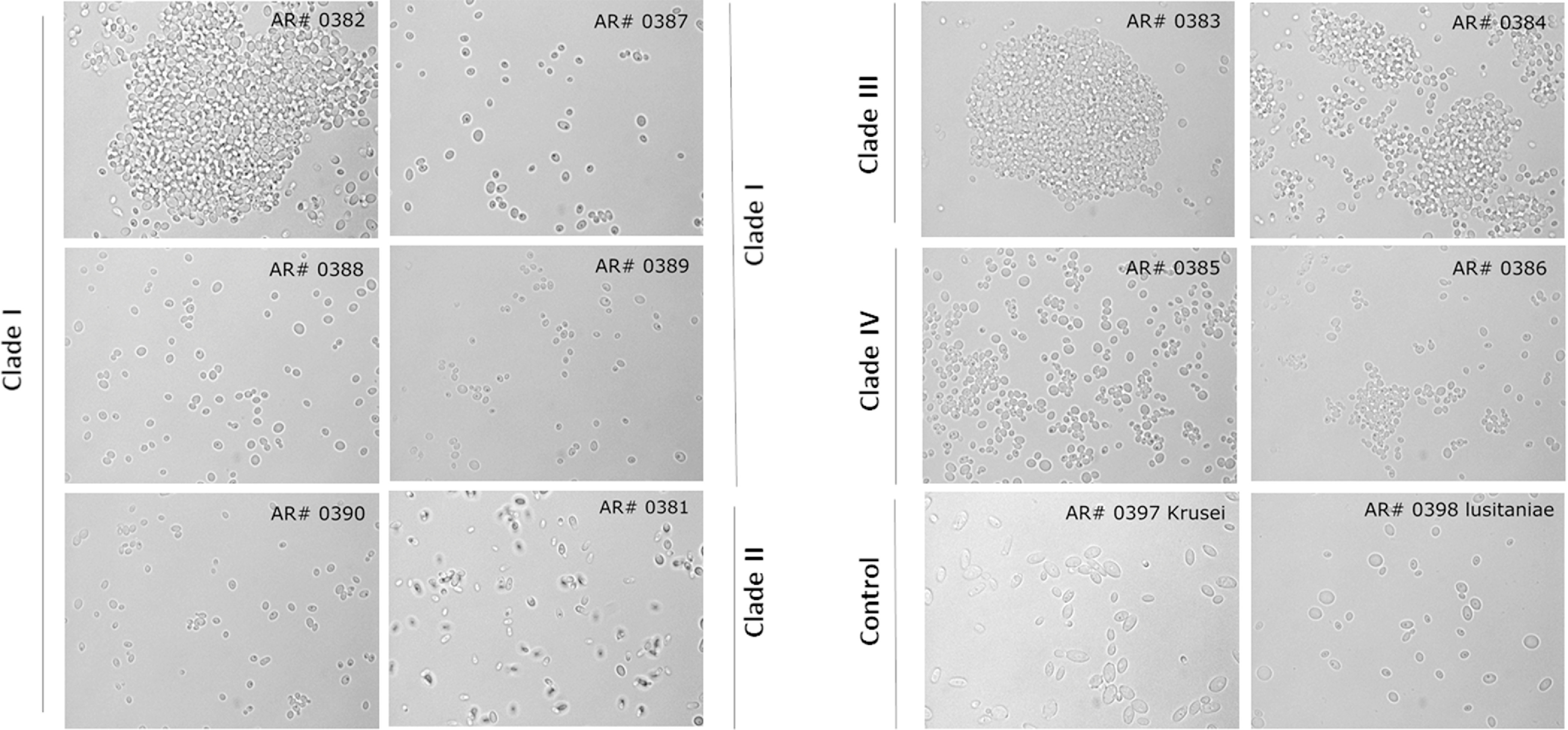
Micrographs of different *C. auris isolates belonging to different clades*. Images taken under a microscope (x100 magnification) for all 10 isolates of *C. auris* belonging to four different clades I-IV in PBS suspensions. *C. krusei* and *C. lusitaniae* were used as controls.

We evaluated the cellular morphology of all *C. auris* isolates using a microscope. Strains from clade III (isolates AR# 0383 and AR# 0384) formed aggregates (Fig. 2), whereas strains from all other clades (except AR#0382 from clade I) were non–aggregate forming.

## Discussion

High virulence and pathogenicity of *C. auris* coupled with reduced susceptibility to antifungals have already caused several outbreaks worldwide.^12^ Various chemical disinfectants such as 27.5% hydrogen peroxide with 5.8% peracetic acid (OxyCide, Ecolab, St Paul, MN) or 10% sodium hypochlorite (Clorox, Oakland, CA), and accelerated hydrogen peroxide (Oxivir TB, Diversey, Charlotte, NC) have shown >5 log_10_ reduction, while other commonly used quaternary ammonium compound–based disinfectants are less effective.^8^ *C. auris* has been shown to persist especially on moist hospital surfaces such as sinks for a prolonged period.^13^ Due to the possibility that some hospital surfaces may be inadvertently missed during manual cleaning combined with the sporadic frequency of manual cleaning events, the addition of more efficient no-touch disinfection devices to the overall cleaning process may be beneficial. We found that *C. auris* isolates are susceptible to UV-C; however, clade differences in susceptibility to UV-C must also be considered. It is known that the complex cell structure and the glucan cell wall layer of eukaryotes render them less susceptible to UV light compared to prokaryotes.^10^ In our study, we observed relatively lower log kill levels for *C. auris* isolates at a high concentrations of 10^6^ cells/mL than have been reported in other studies.^10,11^ These differences in results could be related to 2 factors: First, one study used glass slides instead of steel carriers, and different surfaces and the different spread of inoculum on them might affect the outcome.^11^ Previous studies have shown that the larger the surface on which the inoculum is spread, the higher the kill rate by UV-C.^10^ Second, the intensities of the UV-C bulbs were different based on the manufacturer used between these experiments. Interestingly, when a lower concentration of 10^5^ cells were used on the carriers, we achieved higher log kill, similar to the aforementioned studies. Because the concentration-dependent effect of the UV susceptibility of various clades was not the focus of this study, we have not included these data.

Our data also demonstrate that UV-C exposure had little discernable effect on log kill of isolates belonging to clade III, even after 30 minutes, which exceeded the manufacturer’s recommended UV disinfection protocol. Therefore, shorter duration of UV-C exposure would not be as effective for isolates belonging to clade III compared to the other clades. It is likely that these isolates demonstrate resistance to UV-C because they form aggregates. These aggregates can confer a protective effect and thus prevent the penetration of UV-C light to the core of the aggregate due to a stacking effect. A study of 50 isolates from the South African lineage (clade III) produced similar cell aggregates, which likely aided in biofilm formation.^14,15^ Interestingly, 1 isolate (AR#0382) belonging to clade I, which demonstrated the lowest log kill at 30 minutes, also formed an aggregate. Our findings are consistent with those of Szekely et al,^16^ who found that some isolates of *C. auris* belonging to clade I and almost all belonging to clade III were phenotypically different (ie, formed aggregates) than the other clades.

In conclusion, our findings suggest that it is unlikely that using the same dose and duration of UV would be effective against all isolates of *C. auris* to the same extent. A previous study only used 1 *C. auris* isolate and precluded comparison among different clades.^17^ We acknowledge that even when the disc carriers were placed at a 1.5-m (5-ft) distance perpendicular to the UV device, adequate log kill was not achieved among all the clades studied here at a higher concentration, possibly due to a stacking effect. Therefore, further study is warranted to address this issue. Apart from chemical disinfectants, which have been shown to be efficacious for disinfection, the addition of automated UV-C devices may be complementary and may provide some incremental benefit in achieving superior environmental disinfection. One limitation of this study is that it includes a relatively small number of isolates from each clade. This study generates awareness about the problems of *C. auris* persistence on hospital surfaces and does not provide recommendations for the use of UV devices. Future studies using these UV devices in a real hospital setting where *C. auris* is prevalent may provide additional information that will help control the spread of this pathogen.
